# Supplementary material for: Serological Number for Characterization of Circulating Antibodies
Source: Int J Mol Sci. 2019 Jan 30;20(3):604. doi: 10.3390/ijms20030604 (PMC6387039; doi:10.3390/ijms20030604)
Supplement: Supplementary file 1 [file ijms-20-00604-s001.pdf]

International Journal of *Molecular Sciences*

Supplementary Materials

Serological Number for Characterization of Circulating Antibodies

Andrea Palermo and Alexander Nesterov-Mueller *

Institute of Microstructure Technology, Karlsruhe Institute of Technology (KIT), Eggenstein-Leopoldshafen 76344, Germany; andrea.palermo@posteo.de

* Correspondence: Alexander.Nesterov-Mueller@kit.edu

S1. Immunostaining of Peptide Arrays at Different Serum Dilutions

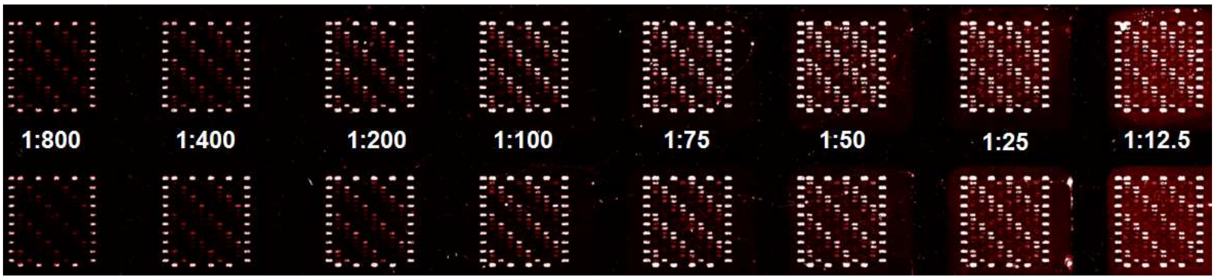

Figure 1. Fluorescent image of the peptide arrays at different dilutions. The white spots in the frames represent reference signals.

S2. Fluorescent Signals of the Peptides from Table 1 versus the Dilution (Circles) and Their Fitting with a Saturation Curve (Red Line) According to Equation (7)

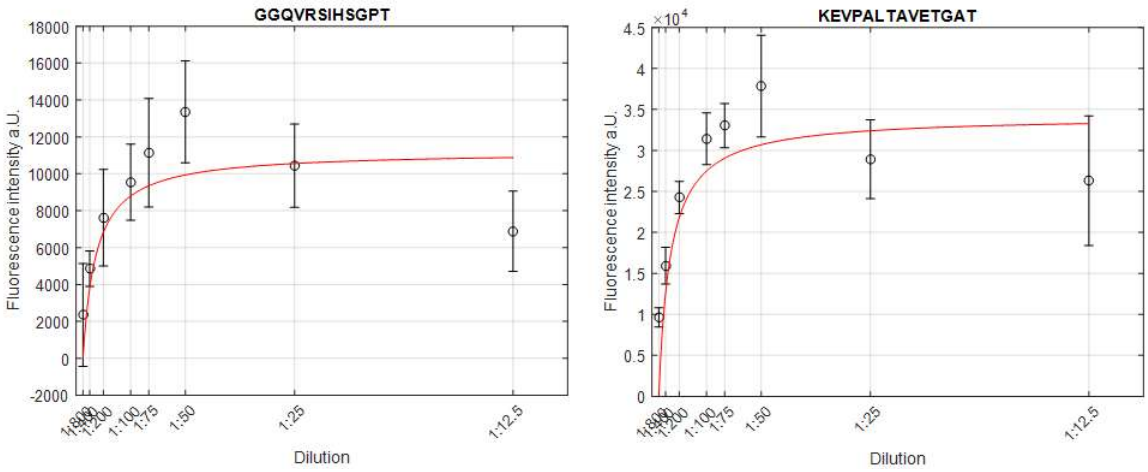

Figure 2. Saturation curves of peptides GGQVRSIHSGPT and KEVPALTAVETGAT.

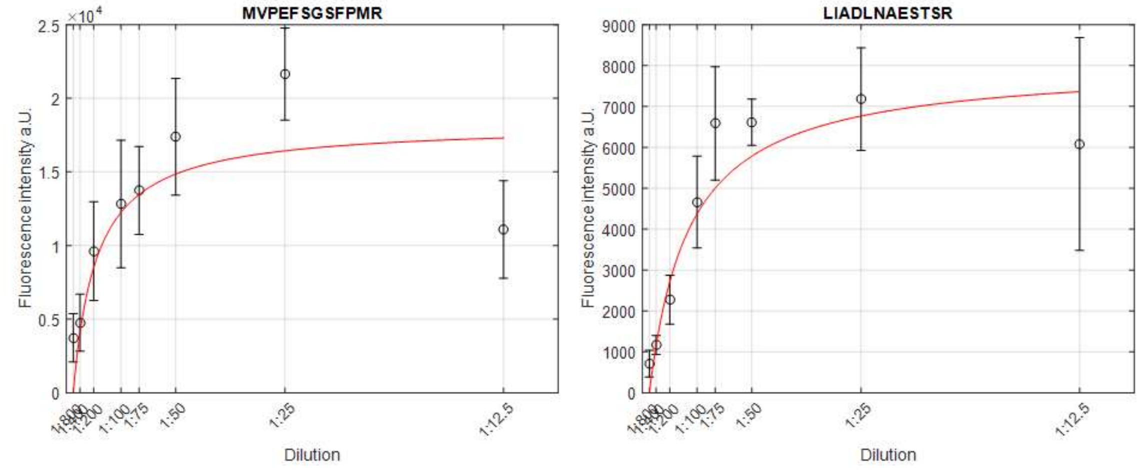

Figure 3. Saturation curves of peptides MVPEFSGSFPMR and LIADLNAESTSR.

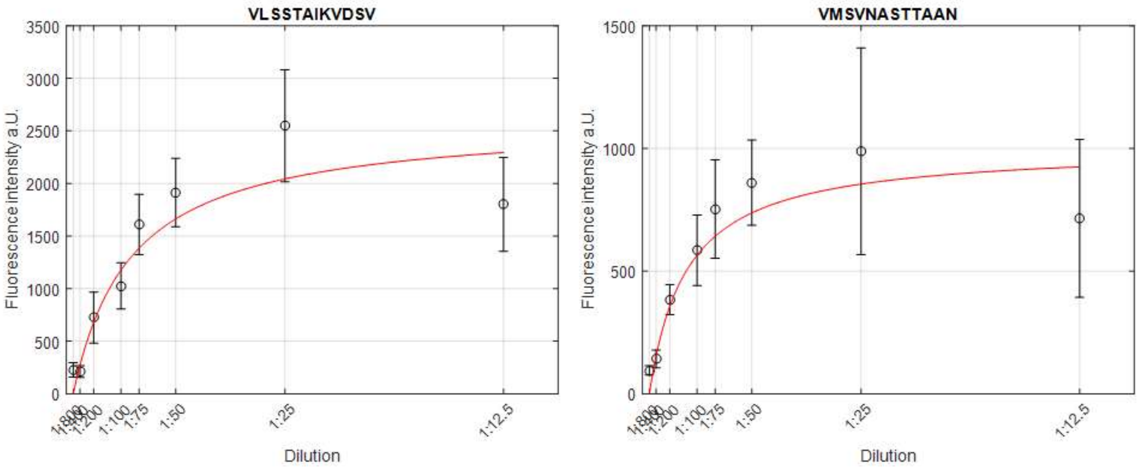

Figure 4. Saturation curves of peptides VLSSTAIKVDSV and VMSVNASTTAAN.

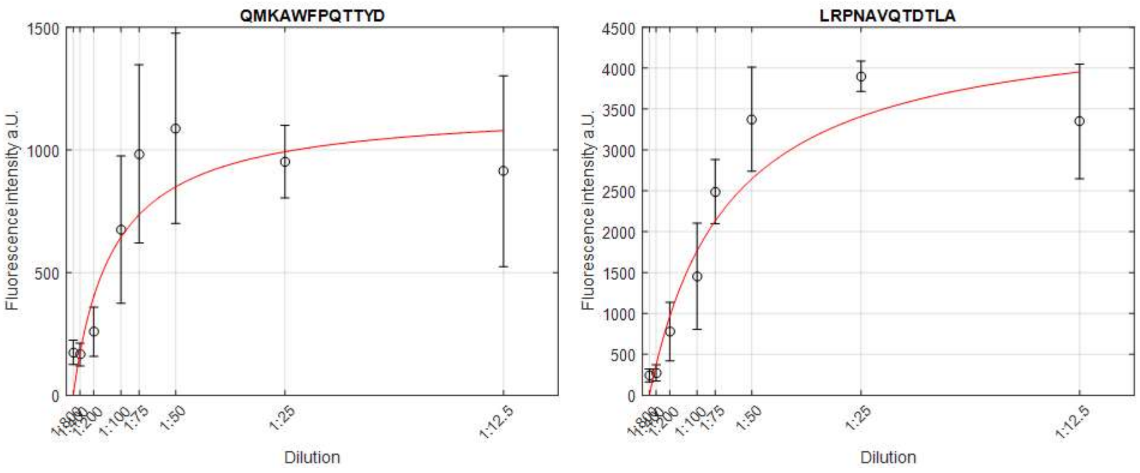

Figure 5. Saturation curves of peptides QMKAWFPQTTYD and LRPNAVQTDTLA.

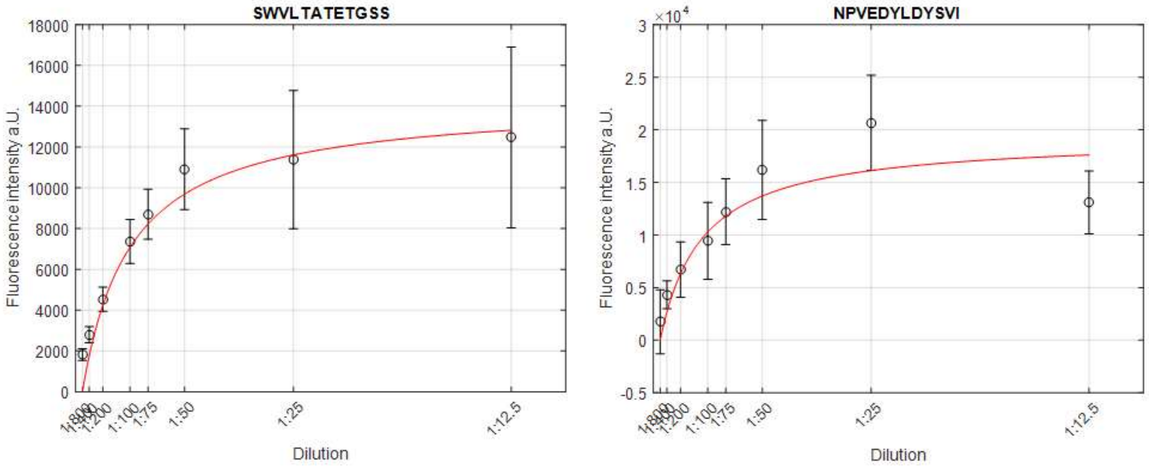

Figure 6. Saturation curves of peptides SWVLTATETGSS and NPVEDYLDYSVI.

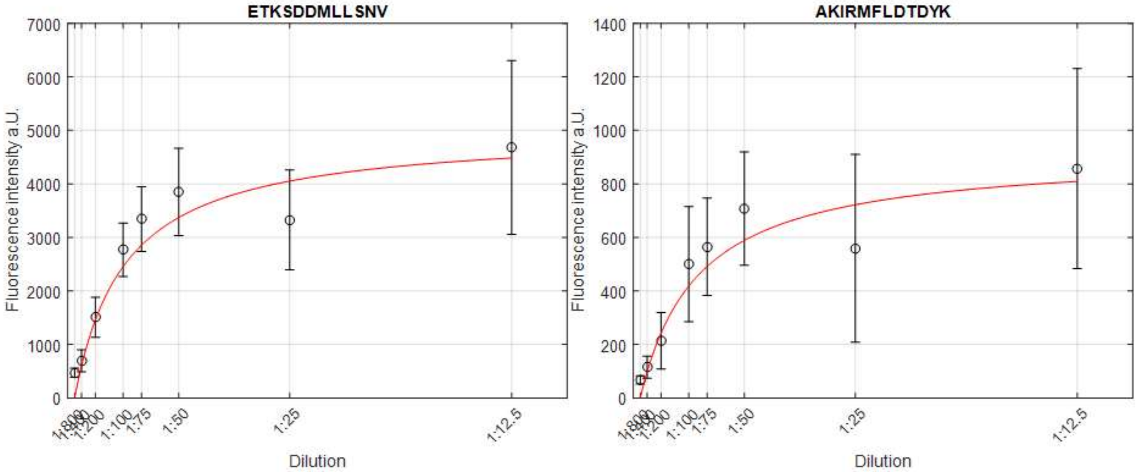

Figure 7. Saturation curves of peptides ETKSDDMLLSNV and AKIRMFLDTDYK.

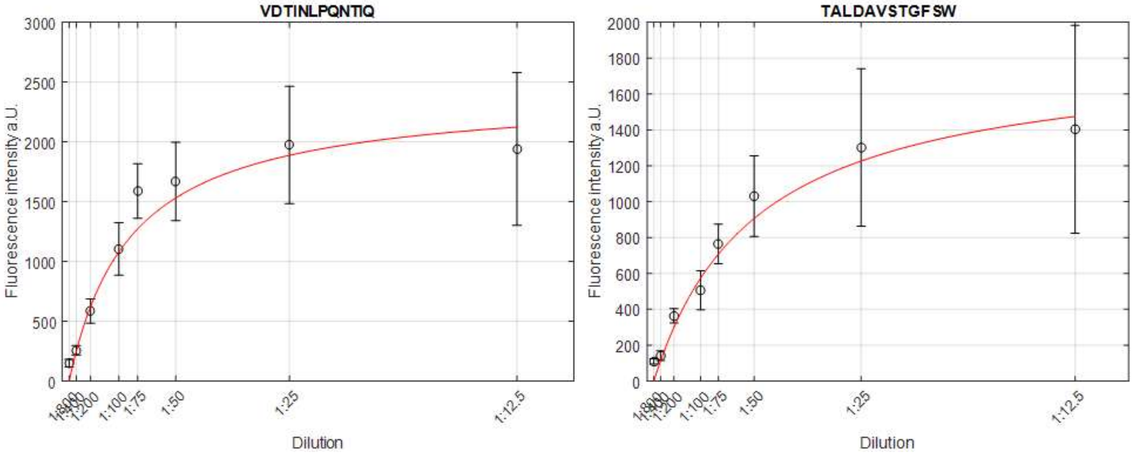

Figure 8. Saturation curves of peptides VDTINLPQNTIQ and TALDAVSTGFSW.

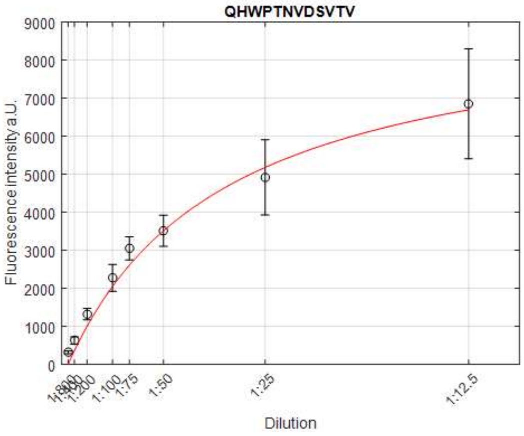

Figure 9. Saturation curves of peptide QHWPTNVDSVTV.

S3. MATLAB Code to Approximate Fluorescent Signals for Peptides (Circles in S2 with a Saturation Curve (Red Line in S2) and to Calculate the *S*-number

MATLAB Function [Result] uses the table with a fluorescent signals at different dilutions from different slides “Tabelle, Slidenr“ to generate the saturation curves and corresponding *S*-numbers.

```
% 1) Import data as table
% 2) Specify variables for dilution used (x_labels), position of x_labels (x_ticks) and slide number (Slidenr)
function [Result]=SerumPlot(x_labels, x_ticks, Table, Slidenr)
% Aqcuire the dimension of the dataset „Table“
List_Dilution=sort(unique(Table.Dilution),'descend');
Count_Dilution=size(List_Dilution,1);
List_Peptide=sort(unique(Table.Peptide));
Count_Peptide=size(List_Peptide, 1);
% Generate model for fitting according to Fobs=(Fmax*C)/(S+C)
g = fittype( @(c, S, x) c*x./(S+x) );
opts = fitoptions( g );
opts.Display = 'Off';
% Generate matrix for data storage
MedianInt=nan(Count_Dilution, Count_Peptide);
Result(1,:)={'ID','Peptide', 'Intensity','Fmax' ,'S', 'rs'};
% Data fitting and plotting
for iPep=1:Count_Peptide
figure(iPep);
% Define datasets for plotting (x, y),
% calculate standard deviation for each peptide and each dilution per slide
for iDil=1:Count_Dilution
MedianInt(iDil,iPep)= median(Table.RedForegroundMedian(Table.Dilution==List_Dilution(iDil) & strcmp(Table.Peptide, List_Peptide(iPep)),:));
MedianStdev(iDil,iPep)= std(Table.RedForegroundMedian(Table.Dilution==List_Dilution(iDil) & strcmp(Table.Peptide, List_Peptide(iPep)),:));
end
% Plotting
YData=MedianInt(:,iPep);
XData=x_ticks;
ZErr=MedianStdev(:,iPep);
errorbar(XData, YData, ZErr, 'ok');
```

```
hold on;
Fmax=max(YData);
Fmin=min(YData);
Xmax=max(XData);
%Define boundaries for fitting. Adjust if necessary
opts.Lower = [0 0];%[Fmin  1];
opts.Upper = [Inf Inf];%[Fmax 1];
opts.StartPoint= [1 1];
lb= [-Inf, -Inf, 0, 0];
ub= [Inf, Inf, 2*Fmax, 2*Xmax];
% Fitting
[fitresult, gof] = fit( XData, YData, g, opts );
plot(linspace(min(XData),max(XData),1000),feval(fitresult,linspace(0,Xmax,1000)),'r')
hold off;
cf=coeffvalues(fitresult);
H=List_Peptide(iPep);
grid on;
% Annotations to the plot
xlabel('Dilution');
set(gca, 'XTick', XData, 'XTickLabelRotation', 45, 'XTickLabel', x_labels)
ylabel('Fluorescence intensity a.U.');
Title_Seq=([H{1}]);
title([ Title_Seq ]);
hold off;
% Save figure
filenamebp=(['Fit_', H{1},Slidenr,'.fig']);
savefig(filenamebp);
hold off;
% Save fit coefficients to 'Result'
Result(iPep,:)={iPep, H{1}, 'Intensity', cf(1), cf(2), gof.rsquare};
end
end
```

© 2019 by the authors. Submitted for possible open access publication under the terms and conditions of the Creative Commons Attribution (CC BY) license (http://creativecommons.org/licenses/by/4.0/).
